# Adapting a *Quercus robur* allometric equation to quantify carbon sequestration rates on the Middle Elbe floodplain

**DOI:** 10.1016/j.mex.2022.101800

**Published:** 2022-07-28

**Authors:** Heather Alyson Shupe, Kai Jensen, Kristin Ludewig

**Affiliations:** Applied Plant Ecology, Institute of Plant Sciences and Microbiology, Universität Hamburg, Ohnhorststraße 18, 22609 Hamburg, Germany

**Keywords:** Carbon sequestration rate, Carbon stock, Quercus robur, Basal area increment (BAI), Diameter at breast height (DBH)

## Abstract

Destructively sampling old Pedunculate oak (*Quercus robur*) trees on the active floodplain of the Middle Elbe to create an allometric equation to estimate carbon stocks (CS) and carbon sequestration rates (CSR) would defeat the purpose of protecting increasingly vulnerable and threatened primeval floodplain forests. To nondestructively estimate CS and CSR, we have adapted a two-parameter allometric equation which uses tree height (H) and diameter at breast height (DBH) (Dik 1984, Zianis et al. 2005) into a 1-parameter equation that requires only DBH to quantify stocks and annual changes in carbon stock (carbon sequestration rates) for individual *Q. robur* trees. The equations have also been adapted to estimate below- and above-ground carbon stocks of individual trees.

The new method has:

• Adapted a 2-parameter *Quercus robur* allometric equation which estimates tree volume to a 1-parameter equation which estimates above and below-ground carbon stock
• Removed the requirement of tree height to reconstruct the carbon stock of trees at an annual timestep
• An almost perfect linear relationship (Pearson R^2^= 0.998) between carbon sequestration rate and basal area increment (BAI)

## Graphical abstract

**Figure:**
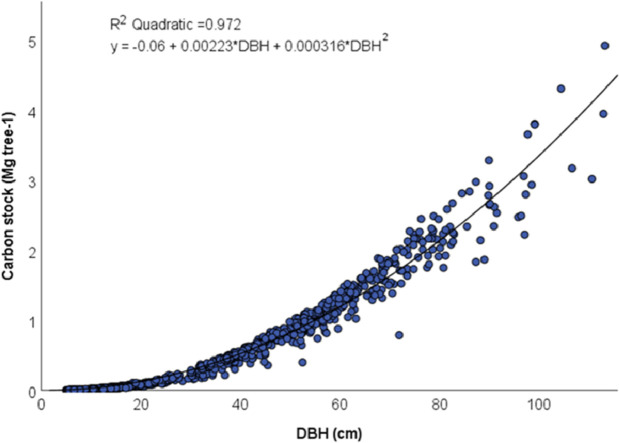

Specifications table

**Table:** 

Subject Area:	Agricultural and Biological Sciences
More specific subject area:	Forestry and Carbon sequestration
Method name:	DBH-based allometric equation of *Quercus robur* for carbon sequestration rate estimation
Name and reference of original method:	Dik [5]. Estimating the wood volume of standing trees in forestry practice. Rijksinstituut voor onderzoek in de bos en landschapsbouw de Dorschkamp, Wageningen. Uitvoerige verslagen (19) 1-114. Zianis et al. [15]. Biomass and Stem Volume Equations for Tree Species in Europe. Silva Fennica Monographs 4:1-63. Shupe et al. [12]. Carbon Stocks of Hardwood Floodplain Forests along the Middle Elbe: The Influence of Forest Age, Structure, Species, and Hydrological Conditions. Water 13. Allometric equation for *Quercus robur* (aboveground trunk and bark volume) developed by [5] and republished by Zianis 2005. Allometric equation (above- and below-ground carbon stock) developed by Shupe 2021.
Resource availability:	N.A.

## Introduction

Destructively sampling old Pedunculate oak (*Quercus robur*) trees on the active floodplain of the Middle Elbe to create an allometric equation for estimating carbon stocks (CS) and carbon sequestration rates (CSR) is not feasible, because it would require cutting old trees in protected and threatened primeval floodplain forests. To nondestructively estimate CS and CSR, we have adapted a two-parameter allometric equation which uses tree height (H) and diameter at breast height (DBH) [5,15] into a 1-parameter equation that requires only DBH to quantify CS and CSR for individual oak trees (Table 1). The original allometric equation by Dik estimates the aboveground volume of tree trunk and bark for *Quercus robur* [5]. This equation was published in the compilation of allometric equations by Zianis as equation #207 in Appendix C [15]. In this study, we adapt this equation to estimate below- and above-ground carbon stocks of individual trees in Mg tree^−1^.

**Table 1:** Equations to estimate volume, carbon stocks (CS), and carbon sequestration rates (CSR) of individual *Quercus robur* trees. Diameter at breast height (DBH) is required for all equations and tree height (H) is required only for the Dik [5] volume and Shupe 2021 CS equation. The Shupe 2021 CS equation estimates below- and above-ground CS by applying three conversion factors to the Dik volume equation. Parameter d is an expansion factor to compute biomass from volume using a species-specific wood density fraction from the Global wood density database [14]. Parameter e is the carbon content [6]. Parameter f is the below-ground carbon estimation [9]. Shupe 2022 CS and CSR parameters a, b, and c are computed using a quadratic fit equation applied to the Shupe 2021 CS estimates of 966 *Q. robur* trees measured in 2018 in the floodplain forests of the middle Elbe. DBH_t_ is the DBH of the tree at the year being analyzed and DBH_t1_ is the DBH of the tree the previous year. Tree ring widths (TRW) of the increment cores are measured at an annual time step and converted into cm.

Source	Output	Units	Equation		Parameters					
		DBH	H		a	b	c	d	e	f
Dik [5] Zianis et al. [15]	Volume (dm^3^)	cm	m	DBH^a^·H^b^·exp(c)	2.00333	0.85925	-2.86353			
Shupe et al. [12]	CS (Mg tree^−1^)	cm	m	(DBH^a^·H^b^·exp(c))*d*e*f	2.00333	0.85925	-2.86353	0.56	0.5	1.3
Shupe [16]	CS (Mg tree^−1^)	cm		a+b*DBH+c*DBH^2^	-0.06	0.00223	0.000316			
Shupe [16]	CSR (Mg tree-1 year-1)	cm		a+b*DBH_t_+c*DBH_t_^2^ - a+b*DBH_t1_+c*DBH_t1_^2^	-0.06	0.00223	0.000316			

Diameter at breast height (DBH) is perceived as the most precise independent variable to estimate tree biomass with allometric equations [4,13]. Past research has estimated CS of trees using allometric equations which require only DBH changes (Köhl et al. 2017). Carbon sequestration rates (CSR) have also been measured using tree cores, allometric equations with DBH, and DBH reconstructions [11]. The removal of height from an allometric model to estimate CS and CSR from DBH is therefore feasible.

## Method

### Estimating carbon stock of trees in 2018

We estimated the aboveground volume of 966 *Quercus robur* trees of several community classes using the Dik equation's two input parameters DBH and H (Table 1). Tree DBH and H were measured in the middle Elbe study region in the winter of 2018/2019 [12]. We converted the volume estimates to above- and below-ground CS estimates with three conversion factors (parameters d, e, and f in Table 1). For the first conversion factor, we multiplied the volume by a species-specific wood density of 0.56 [14] to estimate biomass. Secondly, we multiplied the biomass by 0.5 to estimate carbon content [6]. Finally, we estimated the total biomass (above- and below-ground biomass) by using a root:shoot ratio of 0.3 [9] and therefore multiplied the above-ground biomass estimate by 1.3.

### Creating a one-parameter quadratic equation to estimate carbon stock of *Quercus robur* trees

**Fig. 1:**
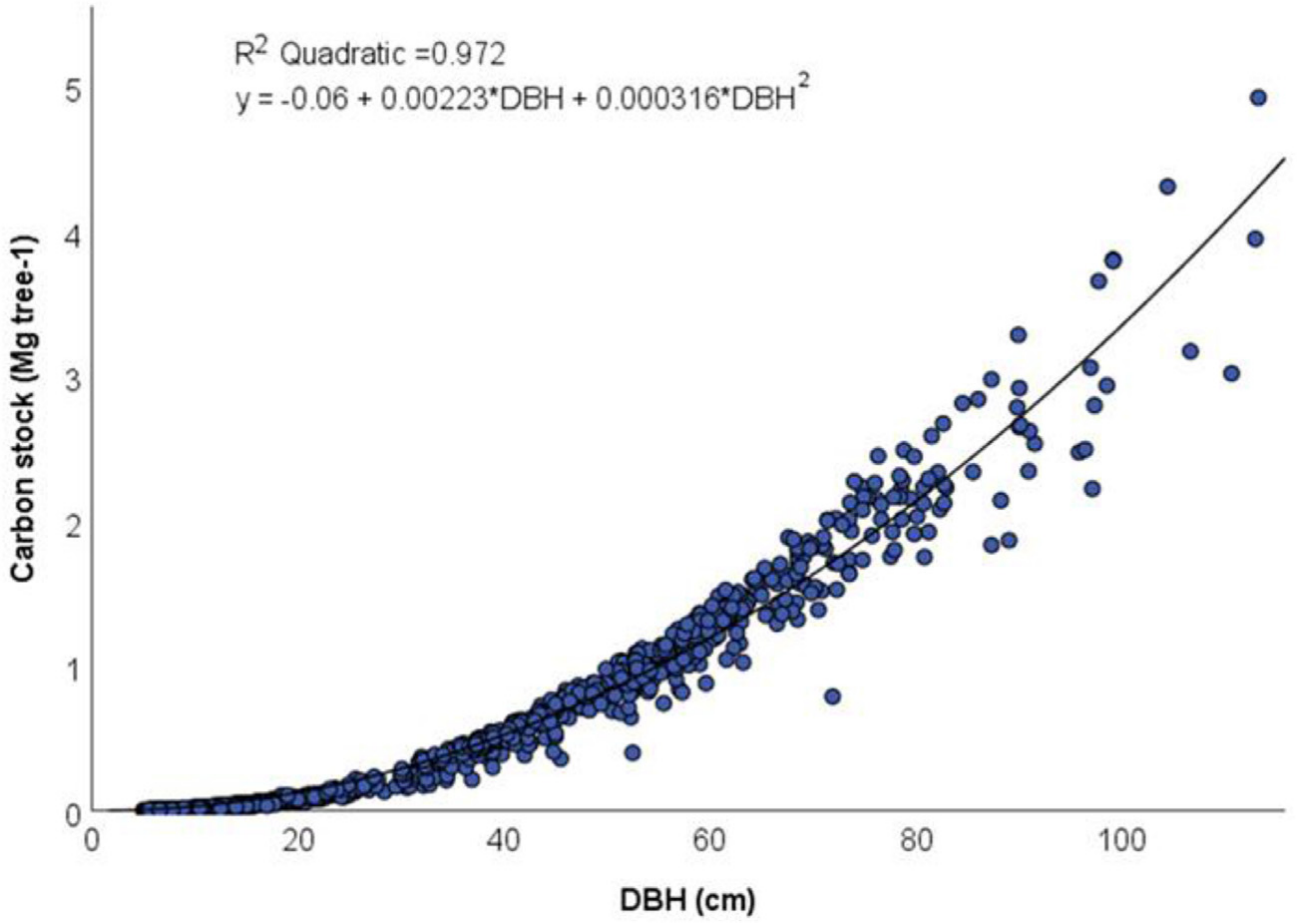
Carbon stocks of individual oak trees (*Quercus robur*) are plotted using the allometric equation from [5] and [15]. A quadratic fit (R^2^ = 0.972) and the developed allometric equation are shown.

After applying the modified Dik equation to the individual trees to estimate CS (Shupe 2021 equation in Table 1), we plotted CS with DBH (see Fig. 1) and applied different lines of best fit. The one-parameter quadratic equation to estimate CS with DBH showed a good Pearson R^2^ of 0.972 and was therefore deemed suitable to reconstruct tree CS at an annual scale without the need to reconstruct tree height back in time.

### Carbon sequestration rate equation

For the purpose of this study, we assume that the CSR of an individual tree is the change in CS from one year to the next. Therefore, when we can estimate the change in the DBH from year to year using tree ring increment core widths, we can recreate the CS of the tree at an annual time step using the developed CS equation, and the change in the CS from year (t) to year (t1) is then the CSR (see Table 1). The DBH of each tree was reconstructed in an annual step-wise fashion using the measured TRW from increment cores and the conventional DBH reconstruction method [2,3]. This method entailed measuring the DBH of each tree at year 2018, subtracting twice the bark width, and incrementally subtracting twice the measured TRW for each year back in time.

## Method validation

Using the dataset of 966 *Q. robur* trees to compare the CS estimate with both DBH and H parameters (Shupe 2021) to the CS estimate with only the DBH parameter, we calculated the root mean square error (RMSE) for all trees and for trees in different DBH size classes. Overall, the RMSE was equal to 1. The greater the DBH, the greater we observed the RMSE to be. Trees with a 5cm ≤ DBH < 35cm had a RMSE of 0.12, trees with a 35cm ≤ DBH < 70cm had a RMSE of 0.97, and trees with a 70cm ≤ DBH < 140 cm had a RMSE of 2.6. Therefore, as DBH increases, the variability between the observed and predicted CS values also increases. Overall, the two models were highly correlated (R^2^ = 0.972).

No destructive sampling of trees was conducted in the protected UNESCO Biosphere Reserve River Landscape Elbe study area. We therefore can validate our CS and CSR equation by comparing the output of the equation with basal area increment (BAI), which is commonly used to compare tree productivity.

**Fig. 2:**
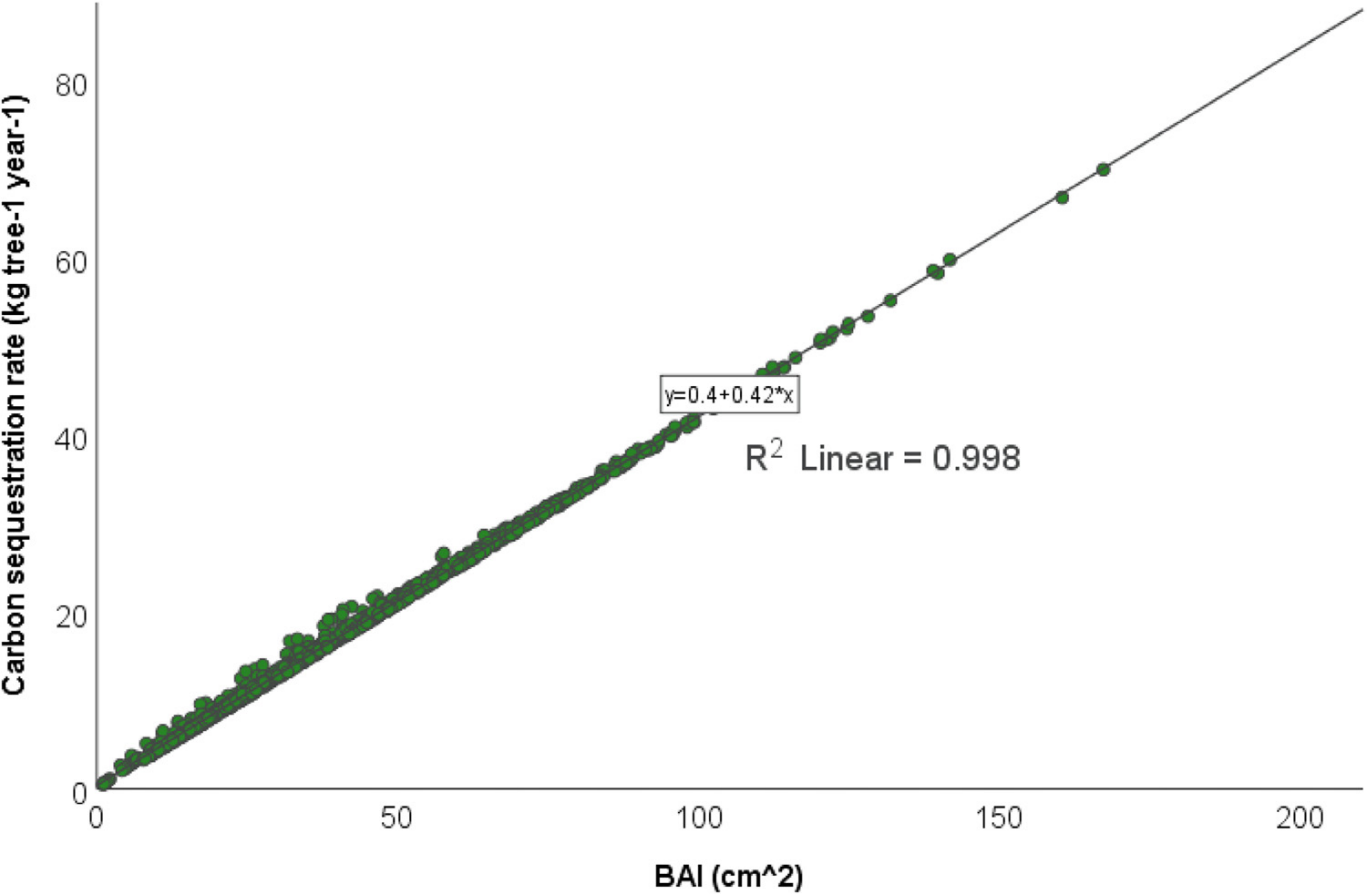
Pearson correlation between basal area increment (BAI) and the estimated total (above- plus below-ground) carbon sequestration rate of *Quercus robur* trees in the Middle Elbe area.

Basal area increment (BAI, Equation 1) and aboveground biomass increment (ABI) have been used in previous dendrochronological studies to compare tree productivity, quantify interannual variability, and improve terrestrial carbon accounting [8]. Additionally, the change in carbon stock estimated with allometric equations and increment core measurements has been used to reconstruct carbon accumulation in forest stands [1]. An allometric equation will never be able to perfectly predict the CS and CSR of every tree because site-specific factors such as competition, soil properties, tree age, and hydrological conditions influence the growth of individual trees [10]. However, here we find that the linear relationship between BAI and our estimated CSR is nearly perfect, with a Pearson R^2^ of 0.998 (Fig. 2). This method is therefore assumed to be suitable for the purpose of non-destructively estimating the average CSR of *Quercus robur* trees in the middle Elbe region.

$$BAI=\pi \left(r_{t}^{2}-r_{t-1}^{2}\right)$$

Equation 1 measures basal area increment (BAI) from the radius (r) of the trees measured at breast height (1.3 m above ground).

The original Dik equation was constructed for *Q. robur* trees in the Netherlands, and we expect that the adapted *Q. robur* equations used here could also be applied to estimate CS and CSR of *Q. robur* in other forests in Northern Europe. However, we do not expect that these equations would be suitable for other tree species, and we would recommend different species-specific equations provided in the literature [15]. There are always going to be differences in forest site factors such as edaphic conditions, hydrological conditions, microclimates, stand management, and stand structure. Climate change can also alter site conditions [7]. Although these factors can influence the allometry of individual trees, it is not feasible to create different equations for trees in each of these conditions because it would require destructive sampling of a large number of trees in each condition. In this case, the protected nature of the trees we study prevented this destructive sampling, and we assume that the estimation for the average *Q. robur* tree is suitable for minimizing overall error at the stand or regional level.

## Declaration of interests

The authors declare that they have no known competing financial interests or personal relationships that could have appeared to influence the work reported in this paper.
